# Data regarding association between serum osteoprotegerin level, numerous of circulating endothelial-derived and mononuclear-derived progenitor cells in patients with metabolic syndrome

**DOI:** 10.1016/j.dib.2016.06.015

**Published:** 2016-06-29

**Authors:** Alexander E. Berezin, Alexander A. Kremzer, Tatyana A. Berezina, Yulia V. Martovitskaya, Elena A. Gronenko

**Affiliations:** a Internal Medicine Department, State Medical University, 26, Mayakovsky av., Zaporozhye UA-69035, Ukraine; b Clinical Pharmacology Department, State Medical University, Zaporozhye, Ukraine; c Private Medical Center “Vita-Center”, Zaporozhye, Ukraine; d Immunology Department, Clinical Laboratory “Dia-Service”, Zaporozhye, Ukraine; e Pathology Bureau, Zaporozhye, Ukraine

**Keywords:** Metabolic syndrome, Osteoprotegerin, Circulating endothelial derived progenitor cells, Mononuclear-derived progenitor cells

## Abstract

Metabolic syndrome (MetS) is defined as cluster of multiple metabolic and cardiovascular (CV) abnormalities included abdominal obesity, high-normal blood pressure, dyslipidaemia, and impaired fasting glucose tolerance that exhibits has a growing prevalence worldwide. We investigated whether an elevated level of osteoprotegerin (OPG) predicts imbalance between different phenotypes of circulating endothelial (EPCs) and mononuclear (MPCs) progenitor cells in MetS patients. We have analyzed data regarding dysmetabolic disorder subjects without known CV disease), as well as with known type two diabetes mellitus. All patients have given their informed written consent for participation in the study. This article contains data on the independent predictors of depletion in numerous of circulating EPCs and MPCs in MetS patients. The data are supplemental to our original research article describing detailed associations of elevated OPG level in MetS patients with numerous of EPCs and MPCs beyond traditional CV risk factors.

**Specifications Table**

**Table:** 

Subject area	*Endocrinology*
More specific subject area	*Metabolic syndrome, progenitor cells*
Type of data	*Tables, figures*
How data was acquired	*Ultrasound, Flow cytometric technique, High-Definition Fluorescence Activated Cell Sorter methodology, ELISA biomarker kits*
Data format	*Analyzed*
Experimental factors	*Conventional cardiovascular risk factors and number of circulating endothelial and mononuclear-derived progenitor cells in metabolic syndrome*
Experimental features	*Serum osteoprotegerin level independently predicts depletion in endothelial and mononuclear-derived progenitor cells beyond classic cardiovascular risk factors in metabolic syndrome patients*
Data source location	Zaporozhye region of Ukraine
Data accessibility	*Within the Data in Brief article*

**Value of the data**

• Previously unreported a predictive value of elevated OPG level in metabolic syndrome patients without known cardiovascular disease on number of circulating endothelial and mononuclear-derived progenitor cells.
• May improve predictive models based on several biomarkers including dyslipidemia, galectin-3, and HOMA-IR in metabolic syndrome patients.
• May stimulate the novel research regarding clinical utility of osteoprotegerin level as predictive biomarker of endothelial dysfunction and cardiovascular events.

## 1 Data

The mean serum level of OPG among entire MetS patients’ cohort was 1142±186 pg/mL vs. 245±75 pg/mL in the healthy individuals (*p*<0.001). Patients with MetS were divided in to two subgroups depended on serum level of OPG using mean value as cutoff point). Subjects with OPG level < 1142 pg/mL and ≥1142 pg/mL were included in cohorts with lower (*n*=18) and higher (*n*=29) OPG level, respectively.

**Fig. 1:**
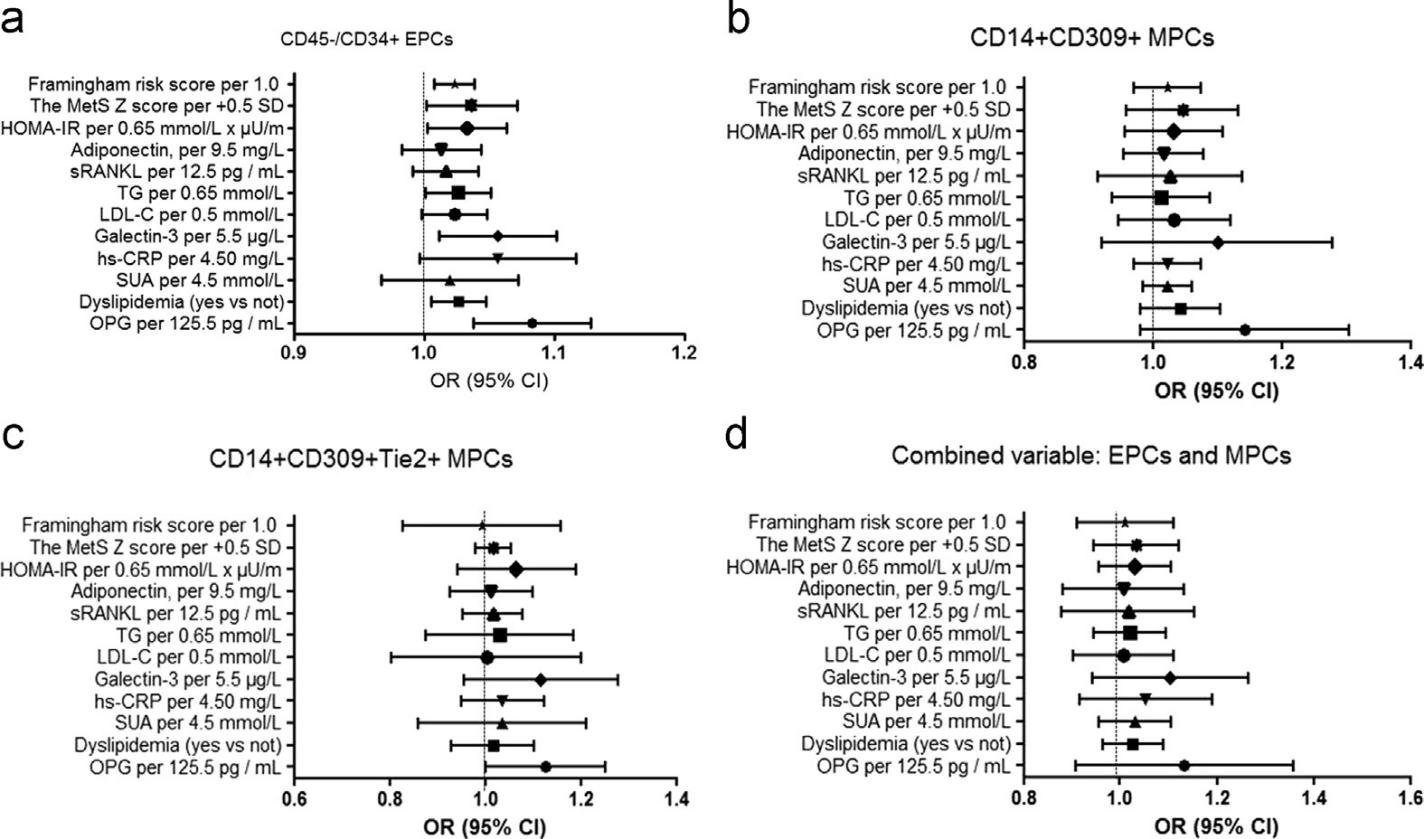
(A) The independent predictors of depletion in numerous of circulating CD45-/CD34+ EPCs. The results of age-, gender-, and BMI-adjusted multivariate logistic regression analysis. (B) The independent predictors of depletion in numerous of circulating CD14^+^CD309^+^ MPCs. The results of age-, gender-, and BMI-adjusted multivariate logistic regression analysis. (C) The independent predictors of depletion in numerous of circulating CD14^+^CD309^+^Tie^2+^ MPCs. The results of age-, gender-, and BMI-adjusted multivariate logistic regression analysis. (D) The independent predictors of depletion in numerous of circulating EPCs and MPCs (combined variables). The results of age-, gender-, and BMI-adjusted multivariate logistic regression analysis.

In multivariate logistic regression analysis we found that OPG, dyslipidemia, galectin-3, and HOMA-IR were independent predictors for depletion in numerous of circulating EPCs and MPCs alone, as well as combined variable: EPCs and MPCs (Fig. 1), whereas the comparison of predictive models based on several biomarkers including dyslipidemia, galectin-3, and HOMA-IR has shown a lack of advantages of these models versus predictive model constructed on OPG alone (Table 2).

## 2 Experimental design, materials and methods

### 2.1 Study design

The results present data regarding 47 patients with MetS who were examined in three our centers between February 2013 and November 2013. We enrolled dysmetabolic disorder subjects without known CV disease including angina pectoris, asymptomatic atherosclerosis (negative contrast-enhanced multispiral tomography angiography), as well as known T2DM. Data received from 35 healthy volunteers were used as a negative control to verify the changes of OPG level and circulating EPCs/MPCs. Healthy individuals were matched with MetS patients on age and sex. All patients have given their informed written consent for participation in the study. Study design was reported recently [1].

MetS was diagnosed based on the National Cholesterol Education Program Adult Treatment Panel III criteria [2].

### 2.2 Anthropometric measurements and smoking status

Anthropometric measurements (body mass, waist circumference, weight, and waist-to-hip ratio) were made using standard procedures. Current smoking was defined as consumption of one cigarette daily for three months.

### 2.3 Echocardiography and Doppler imaging

Transthoracic B-mode echocardiography and Tissue Doppler Imaging were performed according to a conventional procedure on ACUSON scanner (SIEMENS, Germany) using phased probe with modulated frequency of 2.5–5 МHz. Left ventricular end-diastolic and end-systolic volumes, and LVEF were measured by modified Simpson׳s method [3].

LV mass was estimated using formula recommended American Society of Echocardiography׳s Guidelines and Standards Committee and the Chamber Quantification Writing Group [4]. LV hypertrophy (LVH) was defined as a LV mass/body surface area (BSA) ≥96 g/m^2^, for women, and ≥116 g/m^2^, for men [5].

### 2.4 Cardiovascular risk calculation

A 10-year cardiovascular risk for study patients was calculated using the Framingham General Cardiovascular Risk Score (2008) [6] by on-line calculator.

### 2.5 MetS *Z*-score calculation

The MetS *Z* score was calculated using the online calculator (http://mets.health-outcomes-policy.ufl.edu/calculator/) [7].

### 2.6 Calculation of glomerular filtration rate

Glomerular filtration rate (GFR) was calculated with CKD-EPI formula [8].

### 2.7 Measurement of circulating biomarkers

To determine circulating biomarkers, blood samples were collected at baseline in the morning (at 7–8 a.m.) into cooled silicone test tubes wherein 2 mL of 5% Trilon B solution were added. Then they were centrifuged upon permanent cooling at 6000 rpm for 3 min. Plasma was collected and refrigerated immediately to be stored at a temperature −70 °С. Serum OPG, Receptor activator of nuclear factor kappa-B ligand (RANKL), and adiponectin were measured by high-sensitive enzyme-linked immunosorbent assays using commercial kits (R&D Systems GmbH, Wiesbaden-Nordenstadt, Germany) according to the manufacturers’ recommendations.

High-sensitive C-reactive protein (hs-CRP) was measured by commercially available standard kit (R&D Systems GmbH, Wiesbaden-Nordenstadt, Germany).

Fasting insulin level was measured by a double-antibody sandwich immunoassay (Elecsys 1010 analyzer, F. Hoffmann-La Roche Diagnostics, Mannheim, Germany). Insulin resistance was assessed by the homeostasis model assessment for insulin resistance (HOMA-IR) [9] using the following formula:

HOMA-IR (mmol/L×µU/mL)=fasting glucose (mmol/L)×fasting insulin (µU/mL)/22.5

IR was arbitrarily defined as a homeostasis model assessment-IR index (HOMA-IR) value above the 75th percentile of normal glucose tolerance equal 2.45 mmol/L×µU/mL.

Hemoglobin A1c (HbA1c) were determined by high-pressure liquid chromatography method. Concentrations of total cholesterol (TC), cholesterol of high-density lipoproteins (HDL-C), triglycerides (TG), and low-density lipoproteins (LDL-C) were measured by direct enzymatic method (Roche P800 analyzer, Basel, Switzerland).

### 2.8 Blood sampling for measurement f circulating endothelial progenitor cells and mononuclear progenitor cells

Blood samples were received from peripheral vein in blood collection tubes. Each sample contains 75 µL into 1 mL PBS containing 5 µM EDTA (10 µL of 0.5 M stock). To prevent clotting samples were mixed immediately. Peripheral blood mononuclear cells were removed using density gradient centrifugation with Ficoll-Paque (Miltenyi Biotec Inc., Germany). After layer 35 mL of diluted cell suspension over 15 mL of Ficoll-Paque in a 50 mL conical tub all blood samples with anticoagulants (EDTA) have centrifuged at 400×*g* for 30–40 min at 20 °C in a swinging-bucket rotor without brake. The upper layer leaving the mononuclear cell layer was aspirated to prevent a contamination of samples before measurement of real EPCs.

RBCs from samples were removed from the samples using the classic LYSE-WASH protocol. We have the samples washed twice with PBS and fixed immediately.

### 2.9 Determination of circulating EPCs

The flow cytometric technique (FCT) was used for predictably distinguishing circulating cell subsets, which depend on expression of CD45, CD34, CD14, Tie-2, and VEGFR2, using High-Definition Fluorescence Activated Cell Sorter (HD-FACS) methodology [10].

### 2.10 Statistical analysis

Statistical analysis of the results obtained was performed in SPSS system for Windows, Version 22 (SPSS Inc, Chicago, IL, USA). The data were presented as mean (М) and standard deviation (±SD) or 95% confidence interval (CI); as well as median (Ме) and 25–75% interquartile range (IQR). To compare the main parameters of patient cohorts, two-tailed Student *t*-test or Shapiro–Wilk *U*-test were used. To compare categorical variables between groups, Chi2 test (*χ*2) and Fisher F exact test were used. Predictors of EPCs in patients were examined in multivariable regression analysis. C-statistics, integrated discrimination indices (IDI) and net-reclassification improvement (NRI) were utilized for prediction performance analyses. A two-tailed probability value of <0.05 was considered as significant.

## Footnotes

Appendix A Supplementary data associated with this article can be found in the online version at doi:10.1016/j.dib.2016.06.015.
