# Supplementary material for: Data regarding association between serum osteoprotegerin level, numerous of circulating endothelial-derived and mononuclear-derived progenitor cells in patients with metabolic syndrome
Source: Data Brief. 2016 Jun 29;8:717–22. doi: 10.1016/j.dib.2016.06.015 (PMC4949735; doi:10.1016/j.dib.2016.06.015)
Supplement: Supplementary file 1 — Supplementary material [file mmc1.doc]

**Conflicting interests**

Conflicting interests and the full disclosure of any potential relationship with industry are not declared.
